# Label-Free Capacitance-Based Identification of Viruses

**DOI:** 10.1038/srep09809

**Published:** 2015-05-13

**Authors:** Mahmoud Al Ahmad, Farah Mustafa, Lizna M. Ali, Jimsheena V. Karakkat, Tahir A. Rizvi

**Affiliations:** 1 Department of Electrical Engineering, College of Engineering, United Arab Emirates University, Al Ain, UAE; 2 Department of Biochemistry, College of Medicine and Health Sciences, United Arab Emirates University, Al Ain, UAE; 3 Department of Microbiology and Immunology, College of Medicine and Health Sciences United Arab Emirates University, Al Ain, UAE

## Abstract

This study was undertaken to quantitate a single virus suspension in culture medium without any pre-processing. The electrical capacitance per virus particle was used to identify the kind of virus present by measuring the suspension (virus plus medium) capacitance, de-embedding the medium contribution, and dividing by the virus count. The proposed technique is based on finding the single virus effective dielectric constant which is directly related to the virus composition. This value was used to identify the virus type accordingly. Two types of viruses thus tested were further quantified by a biochemical technique to validate the results. Furthermore, non-organic nanoparticles with known concentration and capacitance per particle were identified using the proposed method. The selectivity of the method was demonstrated by performing electrical measurements on a third virus, revealing that the proposed technique is specific and sensitive enough to permit detection of a few hundred virus particles per milliliter within a few minutes.

For efficient control and treatment of viral infections, a rapid method of virus identification (within less than a minute) equipped with continuous monitoring capability is highly desirable. Most of the existing techniques require pre-processing steps which increase the cost and are highly time consuming to perform^1^,^2^. The classification of viruses so far is carried out on the basis of virus particle morphology, viral protein, and/or nucleic acid detection^3^,^4^. Protein-based antibodies are commonly used to capture viruses where each virus type can bind with its corresponding antibody^5^. On the other hand, antibody-functionalized coated nanoparticles could be used to capture the associated viral samples in suspension through specific interactions^6^.

Recently, we have identified and categorized different types of viruses by constructing a concentration-mobility relationship which should be unique for each kind of virus^7^. The type of virus was identified based on a set of electrical parameters which were extracted from the corresponding current-voltage (IV) and capacitance-voltage (CV) profiles. This parameter may become a unique identifier for each virus type and were used for the typing purposes. However, it is possible that two different kinds of viruses with different concentrations could result in the same capacitance and mobility values since what has been measured is a combination of both capacitance and mobility of the whole virus preparation and not per virus particle. Therefore, the current work proposes to find the effective capacitance per virus and correlate it to the virus type.

Viruses are basically complex particles composed of proteins, nucleic acids, and lipids in addition to other minor constituents^8^. Typically, when they are subjected to the propagation of an electrical field, they get polarized. The amount of polarization depends on the virus composition and possibly its interaction with the medium, depending upon its polarity^9^. Hence, any change in the viral electrical capacitance should primarily be due to the difference in the virus particle's intrinsic properties. The virus distribution inside a capacitance structure is shown in Fig. 1(a). In reality, virions are randomly distributed inside the suspension medium. As a model, the virus distribution inside a capacitance structure is considered as two separate zones, Fig. 1(b). It is assumed that the volumes of the virus and medium zones are the same as their respective actual volumes in the suspension. The two zones are modeled as two capacitances in parallel, as shown in Fig. 1(c). Therefore, the suspension capacitance (*C_S_*) (virus plus medium) is calculated from the addition of the two capacitances: bulk (total) viral capacitance (*C_v_*) and mock medium capacitance (*C_m_*):


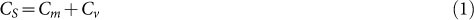
This “parallel model” is based on the observation that the effective capacitance of the virus suspension (virus + medium) was higher than the capacitance of the virus-free mock medium. If the effective capacitance of the suspension was lower than the reference, then a “series model” would have been considered.

De-embedding the relative capacitance of the mock medium from that of the suspension (virus plus medium) starts with virus volume determination which can be computed from the concentration measurements. Next, the proposed technique computes the relative medium capacitance by quantifying the virus counts inside the suspension structure, and determining its volume as well as that of the medium (since the dielectric constant of the medium is known). The corresponding average virion capacitance (*C_vi_*) can thus be computed as follows:


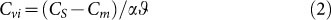
where *α* is the concentration of the virus particle inside the relevant suspension medium and 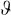 is the volume of the capacitance structure. It is worth pointing out that the method presented here is applicable only to “electrically-polarizable” virus particles when they are suspended in a “conductive medium”. Non-polarizable virus particles cannot be detected by this method.

It is also important to note that we cannot construct a parallel plate capacitor with single virus particles as its dielectric material. Our dielectric materials are either the “mock medium” (virus-free medium) or the virus suspension (virus plus medium). To probe single virus particles is quite expensive and requires dedicated tools and well-trained professional researchers. Additionally, such a process requires heavy sample preparation and single virus particle isolation. Our present technique overcomes this handicap by utilizing a conductive substance such as the liquid medium to pass the current through the virus particles and get them charged. Employing this methodology, we could by-pass the need for going through these cumbersome procedures. Thus, the methodology presented here is a lot more superior when compared to the other state-of-the-art techniques, in addition to being quick, simple and easy to handle, not to mention, very economical.

## Results

**Figure 2:**
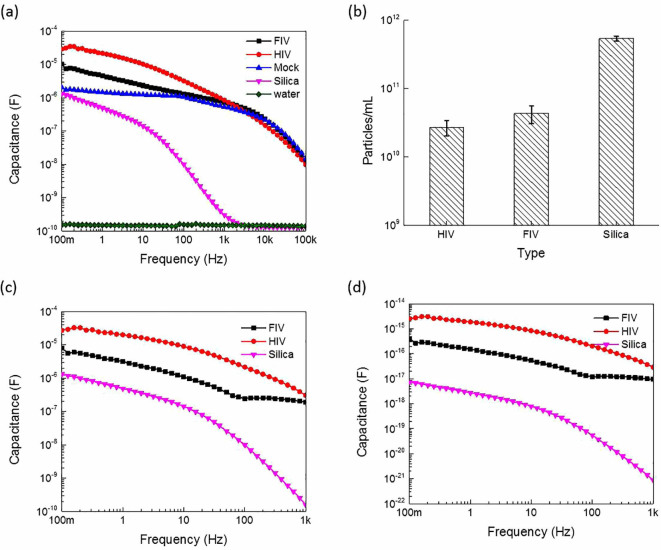
Capacitance and concentration measurements of FIV, HIV, and silica nanoparticles: (a) suspension (virus plus medium) capacitance versus frequency for FIV, HIV, silica nanoparticles along with the relative mock medium, (b) concentration of the involved materials as estimated by the more classical biochemical and spectroscopic techniques, (c) the corresponding ensemble (total or bulk) capacitance, and (d) the capacitance per particle of the tested materials. Error bars represent the standard deviations.

To demonstrate the concept and operational principle of the proposed technique, two different viruses, feline immunodeficiency virus (FIV) and human immunodeficiency virus (HIV)^10^,^11^,^12^ were tested and analyzed in this study. These two viruses belong to the lentivirus group of retroviruses and are of close morphology and dimension (approximately of 110–150 nm)^12^,^13^. In parallel, non-organic silica nanoparticles were used as a control that had similar size and dimension, but a different (inorganic) composition. The mock control medium for the viruses was Dulbecco's Modified Eagle Medium (DMEM) supplemented with 10% fetal bovine serum and appropriate antibiotics, whereas water served as the medium control for the silica nanoparticles. The two viruses were prepared using a calcium phosphate transfection technique reported earlier and described in Methods^7^. The electrical measurements were performed by loading different virus suspensions inside an open-ended coaxial cable and the electrical measurements were achieved using the Gamry 3000 instrument (USA) that has a wide range of electrical measurement capabilities. The virus suspension capacitance measurements (*C_S_*) (virus plus medium) relative to the frequency are shown in Fig. 2(a). It was observed that the virus capacitance profiles (behavior of the virus suspension capacitance) followed the same frequency behavior as that of the mock medium and could be distinguished from the mock in the frequency range between 100 mHz and 1 kHz. Thereafter, the capacitance profiles of the two viruses and mock merged. Thus, all subsequent measurements were done at a frequency range of up to 1 kHz to ensure that distinct viral capacitance profiles could be obtained from those of the mock. On the other hand, the measured capacitance of the silica nanoparticles that have an inorganic nature did not exhibit the same frequency behavior as that of its corresponding medium, but the two profiles still merged after the 1 kHz range, confirming our earlier observation that we should limit our frequency range to between 100 mHz-1 kHz. Since the measurement setup included cables and connectors which affect and dominate the frequency response at 5 KHz onwards; therefore, only the low frequency range was considered in the calculations.

The concentration measurements were performed and the results are shown in Fig. 2(b). The QuickTiter™ Lentivirus Quantitation Kit (Cell Biolabs Inc., San Diego, CA USA) was used to quantify the FIV and HIV loads; it revealed virus titers of 2.0–3.2 × 10^10^ and 3.6–6.2 × 10^10^ for HIV and FIV, respectively^7^. The silica nanoparticles (obtained from *Nanocomposix,* USA*)* with a diameter of 120 nm and adjusted to a concentration of 2.4–5.4 × 10^11^, were suspended in water and their suspension concentrations were measured using the more classical approach of the Agilent 8453 ultraviolet/visible spectrophotometry (UVIS).

The effective (total or bulk) capacitance shown in Fig. 2(c) was extracted by subtracting the relative medium capacitance from the total. This was followed by calculating the corresponding virion capacitance per particle using equation (2) and is shown in Fig. 2(d). The capacitance profiles in the two figures were identical except for the relative capacitance scales. Furthermore, Fig. 2(d) shows that the corresponding extracted capacitance of HIV single virion was higher than the FIV and silica nanoparticles by approximately 10 and 1000 folds, respectively, revealing the “intrinsic” electrical behavior of the two different virus particles that were distinct. The capacitance per single virus particle depends strongly upon lipid and protein content as well as the virus envelope properties which could be influenced by the medium pH. Therefore, the de-embedding step actually plays an important role in the normalization of such influences, eliminating any relative change in pH, polarity and composition of the virus suspension. Thus, the coating of different kind of viruses with the same envelope protein (vesicular stomatitis virus envelope G; VSV-G Env)^7^ and lipid bilayer (from the producer 293T cells) under the same preparation conditions along with the de-embedding step ensured the exclusion of any further mutual interactions between the different kind of viruses and the medium, resulting in normalization of all measurements.

**Figure 3:**
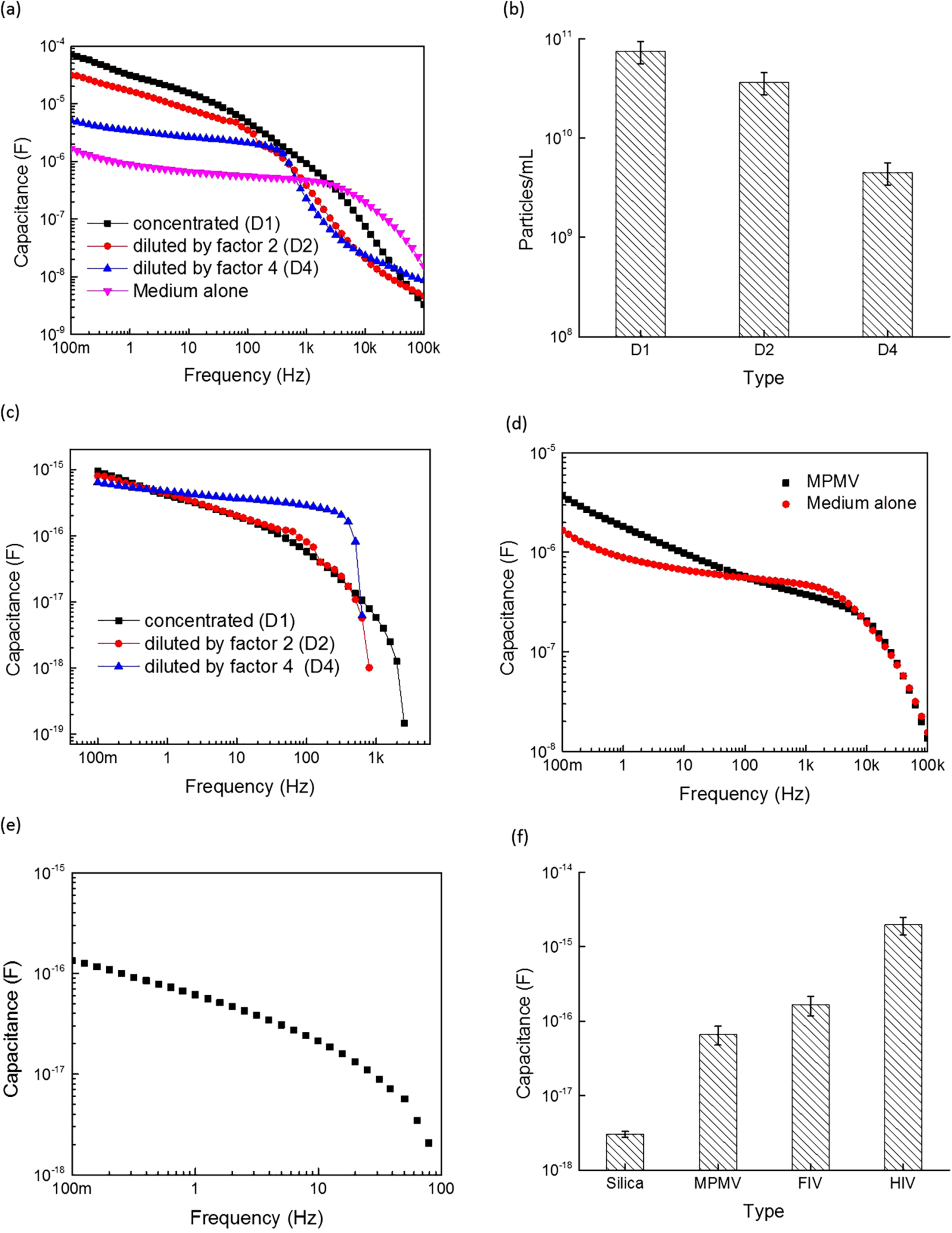
Capacitance and concentration analysis of viruses and nanoparticles: (a) Measured capacitance values of concentrated (D1) and diluted (D2 = two-folds and D4 = four-folds) HIV samples. (b) Measured concentrations corresponding to the capacitance observed in the first panel. (c) Capacitance per HIV virus particle versus frequency for the corresponding concentrated and diluted virion capacitance. (d) Capacitance measurements versus frequency of MPMV virus and its suspension mock medium, (e) MPMV single virus capacitance, and (f) the extracted capacitance per particle at 100 mHz for different material types. Error bars represent the standard deviations.

To further analyze the electrical properties of the organic virus particles, the HIV virus stock was diluted by a factor of 2 and 4 using the mock medium. The capacitance versus frequency of the concentrated and diluted virus stocks were then measured compared to the mock control. As can be observed from Fig. 3(a), the more concentrated the virus particle suspension, the higher the observed effective capacitance values. The electrically-measured corresponding virus concentrations (particles/ml) are summarized in Fig. 3(b) and showed a proportional dilution response. The extracted virion capacitances for the mentioned dilutions are depicted in Fig. 3(c) which showed almost a smooth behavior of virion capacitance in the mentioned frequency, suggesting a lack of electrical interference from possible interaction with light or any other possible sources for cross-talk such as other equipments in the lab.

The effective capacitance measured was composed of two types of capacitances added together - the main virus single particle capacitance (self-capacitance) and the virus-virus capacitance through the conductive medium (mutual-capacitance). The virus-virus capacitance depends on the virus particle size and the distance between them. However, the motion of the virus particles inside the suspension exhibits a random Brownian motion, which is unpredictable and therefore could not be used to measure the distance between the individual particles. Nevertheless, if the distance between two adjacent virus particles are larger than the virus diameter, then this mutual capacitance lends itself to an open circuit capacitance; i.e., has no considerable influence on the effective capacitance, it therefore could be ignored.

Next, another type of virus was examined to demonstrate the selectivity of the proposed technique. Fig. 3(d) shows the total (bulk) capacitance for both the Mason-Pfizer monkey virus (MPMV) and its corresponding mock medium. Similar to what was observed for FIV and HIV, the capacitance profile was distinguishable between MPMV and the mock media in the frequency range below 100 Hz. Similarly, the extracted MPMV single virus particle capacitance is depicted in Fig. 3(e) and showed a smooth profile, similar to FIV and HIV. The single particle capacitance values of the three viruses (HIV, FIV and MPMV) along with the non-organic silica particles are summarized in Fig. 3(f). Comparing the capacitance of the three viruses revealed that each type of virus could be identified from its corresponding eigen virion capacitance; i.e., specific capacitance “signatures” for the three different viruses, which could then be attributed solely to the precise nature of the respective viral particles. These observations strongly suggest that the “capacitance per virus particle” parameter can be used to type different viruses. These signatures can be further narrowed by additional error optimization which can be achieved by optimizing the dimension of the capacitance structure (the coaxial adaptor) from millimeter to micrometer range.

**Figure 4:**
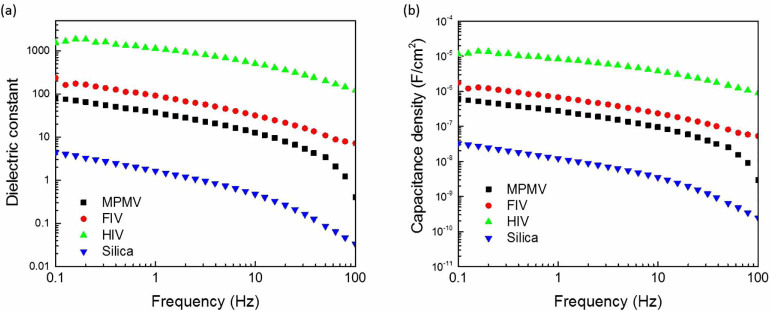
Computed (a) dielectric constant and (b) capacitance density of different type of materials used in this study.

Finally, the dielectric constant and the capacitance density of the viruses under study along with silica nanoparticles were extracted from the isolated sphere capacitance condition^14^ using the equation (3) below and are illustrated in Fig. 4(a) and (b):


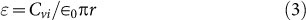
Where: *∈*_0_, *ε* and *r* are the free space dielectric constant, the relative dielectric constant and the radius of the spherical particle, respectively. The capacitance densities per cm^2^ were computed by dividing the capacitance per particle over its surface area.

To exclude the geometry effect of the capacitance structure, it was prudent to identify the virus particle based on the dielectric constant rather than the capacitance itself. The dielectric constant is directly related to the particle composition. By default, each virus is unique with its own protein/nucleic acid composition. That is what makes its individual capacitance unique. Therefore, the effective dielectric constant rather than the capacitance structure of the virus was used for identification of the virus type. Moreover, with the de-embedding process, the geometry of the assay would not affect the capacitance per particle measurements, although the geometry will affect the limit of detection.

The dielectric constant profiles up to 100 Hz versus frequency of all the materials tested showed a smooth behavior (Fig. 4(a)). The effective dielectric constants of the three viruses were higher than the silica particles. The charging of the organic particles (virions) was strongly affected by polarity of the control medium due to the interactions that occur between the particles and the surrounding medium; whereas, the inorganic particles (silica) were not affected by the surrounding medium significantly. In case of the organic suspensions, more charges accumulated on the particle surface due to the possibility of ionic interactions because of their compositions (Fig. 4(b)). On the other hand, the charge accumulated on the inorganic silica particles was highly dependent on their dielectric constant since the strength of their ionic interactions with the medium was weak (Fig. 4(b)).

The presented model accounts for possible virus-virus particle interactions which are by default represented by the cumulative effective capacitance. In case of the co-existence of different types of virus particles in the same suspension (which is not the case in our study), the interactions should be re-modeled and considered specifically.

## Discussion

In our earlier work, we have shown that different virus particles can be ascribed a “signature” value based on their electrical characterization parameters; however, since these parameters were based on “bulk” and not “per particle” values, it was possible that two different viruses could end up with the same value due to changes in their concentrations. Therefore, this study was undertaken to test whether signature sequences particular to a specific virus particle could be obtained using electrical methods on a per particle rather than bulk bases. Towards this end, two related viruses, FIV and HIV, were chosen to demonstrate the sensitivity of the current method. These viruses belong to the lentivirus group of retroviruses^12^. On the other hand, the selectivity of the proposed technique was validated by testing non-organic silica nanoparticles with similar size and concentration to FIV and HIV, as well as a more distantly related retrovirus, MPMV^12^,^15^. In comparison to FIV and HIV, MPMV has a similar size and morphology, but is a simple retrovirus with only the three canonical genes for virus replication. Additionally, the virion assembly processes of FIV and HIV are distinct from that of MPMV. FIV/HIV exhibit type C morphology and can only form extracellular virus particles upon budding from the cell membrane, while MPMV is a type D retrovirus that forms intracellular as well as extracellular virus particles^12^,^16^.

The data presented in this study have been compared with what has been reported so far in literature. For example, Gomila and colleagues recently attempted to extract the dielectric constants of different types of nanoparticles of similar shapes and sizes in the same order of magnitudes, including polystyrene, silicon oxide, and aluminum oxide using electron force microscopy^17^. They observed that the three types of particles could be distinguished based on their dielectric constants which were in the range of 2.6 ± 0.20 for polystyrene, 4.22 ± 0.32 for silicon oxide, and 9.24 ± 0.60 for aluminum oxide. Their data were taken by applying 4 V at 1 kHz, with a scanning speed of 1 s per line, thus achieving high electrical resolution and capabilities of calibration to exclude the cable probing effect. In comparison, our low frequency silica dielectric constant assumed a value of 4 which is close to their reported values (Fig. 4(a)). Similarly, MacCuspie et al. measured capacitance density of five types of viruses by AC capacitance scanning probe microscopy^18^. By comparing our measured capacitance densities and the reported values by MacCuspie et al.^18^, the values presented in this study are in the observed range (between 10^−5^ and 10^−6^ F/cm^2^) as indicated in Fig. 4(b). Hence our findings corroborate well and are in good agreement with earlier published observations.

The accuracy and reproducibility of the current approach has been validated by repeating both the capacitance and concentration measurements for multiple virus stocks prepared at different times. The virus count originally was determined using a biochemical method that measures the viral nucleic acid in a suspension. The capacitance measurement was of high-precision, up to zepto-Farad (10^−21^). Furthermore, the sensitivity of the method was evaluated by diluting a known concentration of the virus with the corresponding mock control medium. The detection limit was observed to be as low as hundreds of particles per milliliter, as previously reported^7^. Hence, these data reveal that the present technique is sufficiently sensitive to allow the determination of virus particles during the early stage of infection.

By combining the virus quantification technique presented earlier^7^ and the current proposed methodology, it should result in a paradigm shift in label-free virus quantification and identification. The two methodologies together should allow one to quantify and identify any polarizable virus electrically in the fastest and cheapest way possible without the need of labeling or any preprocessing steps. In spite of the simplicity of the presented technique, when compared with other complicated and time consuming techniques, this method proves its ability and capability to detect and identify any type of virus particle in a suspension. Therefore, it is expected that this method will accelerate the discovery of fast and efficient label-free characterization and identification of different viruses.

## Methods

### Electrical characterization methods

Gamry reference 3000 instrument (Gamry/USA) which is used to conduct electrical measurements, is a high performance, high current USB potentiostat which has 11 current ranges from 3 amps to 300 picoamps and a compliance voltage reaching to 32 volts. The measurements from the Reference 3000 can perform in a frequency range from 10 μHz to 1 MHz since it is equipped with on-board electronics for electrochemical impedance spectroscopy. For the current experiment, applied oscillation voltage of 15 mVpp was used for impedance measurement in the range of 100 mHz to 100 KHz. Earlier measurements at a high frequency from 1 MHz up to 13.5 GHz for different types of viruses (FIV and HIV), helped us to choose this frequency range. Significant identification record for different virus frequency response was obtained at a range of 100 mHz to 100 KHz, which simplified the procedure further to achieve specific 'signatures' that facilitated identifying and characterizing the different viruses and also investigating the feasibility of titer quantitation using the current method. The radiofrequency generators of the Reference 3000 are capable of producing power signals at different frequencies and also help to measure current voltage, capacitance voltage, polarization in addition to charging/discharging profiles with the ability to change different parameters. The calibration of the system was performed with the aid of manufacture-provided calibration kit*.* In a characteristic calibration, the measurement reference plane moves to the end of the test cables, resulting in the exclusion of effect of loses and phase shifts followed by the addition of noise to the signal measured. Different sets of electrical measurements were conducted after loading the suspension inside an open-ended coaxial cable. To minimize the effect on measurements of interest as detailed above, the self-resonance frequency of the coaxial cables was ensured to be above 100 MHz.

### Limit of detection, sensitivity and accuracy

To determine the limit of detection and sensitivity, a known quantity of the virus was diluted to different concentrations ranging from 10^2^–10^11^ virus particle/ml in its relevant mock free medium. Viral capacitance over this range was measured and then the medium were de-embedded. When the diluted capacitance values were plotted as a function of the quantity of the virus, it exhibited a linear relationship with the intercept not significantly deviating from zero, as previously reported^7^. Thus, the limit of detection was excellent with several hundred virus particles per ml. This also confirms the high accuracy of the presented method. It is worth to add that the smaller the capacitance structure, the higher the detection limit. Furthermore, the only foreseen limitation of this assay resides with the ability of the virus particles under test and the suspension medium to be “polarizable”, as mentioned earlier. Non-polarizable virus particles cannot be detected by this method.

To further establish the “specificity” of the current method, in addition to the two viruses belonging to the lentivirus group namely FIV and HIV, another more distantly related retrovirus was also tested by this methodology: MPMV. Viral particles counts were measured by the previous electrical proposed method in^7^.

The speed, accuracy and reproducibility of the current technique has been checked by repeating the electrical measurements against multiple virus stocks prepared at different times; i.e., all the measurements have been conducted within the mentioned specific frequency range and applied bias voltages. The accuracy of the presented method is comparable with other conventional techniques and may in fact be better in the lower range of detection.

### Electrical parameters extraction algorithm

An algorithm was developed in our previous study to extract electrical parameters of bulk virus preparations when charged^7^. This algorithm was developed based on the assumption that virus particles are modeled as impurities in a semiconducting material and was subsequently validated. Thus, the same algorithm was used in this study to further analyze the electrical behavior of viruses on a per particle basis, as explained in the text. Details of the algorithm can be reviewed in reference 7.

### Adaptor geometry and specifications

Capacitance measurements were conducted in a coaxial adaptor connected to coaxial cables. The structure of the coaxial adaptor comprised of inner and outer conductors with dimensions of 2 and 5 mm, respectively, and a length of 7mm. The control or viral suspension media were loaded into the adaptor serving as the dielectric material. The advantage of the coaxial topology is that the radio frequency signal and the electrostatic field propagations are confined and protected from outside interferences and the signals do not escape space between inner and outer conductors^19^.

### Virus culture, sample preparation and biochemical virus quantitation test

The virus particles used in this study were produced using a genetic *trans* complementation strategy that is routinely used in the laboratory^20^,^21^,^22^ details of which are provided in reference 7. Briefly, the virus particles were produced in human 293T cells by transfecting three individual expression plasmids that produced different parts of the virus particles. This included a plasmid that expresses the viral structural genes (MB22 for FIV, CMVΔR8.2 for HIV-1, and TR301 for MPMV) to form the retroviral particles that could encapsulate the respective retroviral genomes expressed from the plasmids TR394 for FIV, MB58 for HIV-1, and SJ2 for MPMV. A third plasmid expressing the envelope expression plasmid (MD.G) of the vesicular stomatitis virus envelope G (VSV-G) was used to coat the three virus particle surfaces. The three sets of plasmids for each virus were co-transfected into 293T producer cells that released the respected viruses into the tissue culture medium used to culture the cells: Dulbecco's modified Eagle medium (DMEM) supplemented with 10% fetal bovine serum (FBS), penicillin, streptomycin, and gentamycin antibiotics. A parallel set of transfections were carried out using a control plasmid without any viral genes as the “mock” transfected sample. This sample underwent all the steps of the transfection process as the test samples and therefore could serve to normalize for any pH fluctuations or redox differences encountered during the virus preparation process. It also served as the control to “de-embed” the effect of media on the measured capacitance of the virus suspensions.

The virus particles thus produced were then used for further electrical signature parameters. The number of FIV and HIV lentiviral particles released in the tissue culture media were estimated by using QuickTiter™ Lentivirus Quantitation Kit (Cell Biolabs Inc., San Diego, CA USA) using manufacturer's directions. This assay is valuable over other antibody-based methods since both FIV and HIV virus particles can be measured using the same method (measurement of viral nucleic acid content) thereby nullifying any difference in specificity and sensitivity that may arise when different assays are used. Briefly, the mock or viral supernatants obtained from transfected cultures were centrifuged (300 × g for 5 minutes at 4°C) and filtered through a 0.2 micron filter to remove any cellular debris. The clarified supernatants thus obtained were digested with nucleases to completely get rid of any residual nucleic acids from ruptured cells. Finally, the virions were lysed and the viral RNA thus released from virions was detected using a CyQuant® GR RNA binding dye using the Perkin Elmer VICTOR™ X3 Multilabel Plate Reader using a 485/538 nm filter set. The RNA standard provided in the kit for quantification provided a linear curve in the range of 1–1000 ng of lentiviral RNA which was used for the quantitation of FIV and HIV RNAs. The values thus obtained were used to determine viral titers (virus particles/ml) as described in the kit.

## Author Contributions

MA conceived the concept and performed the electrical experimental work. MA, FM, and TAR supervised the project and contributed towards writing and editing the manuscript. JVK and LMA made the virus preparations. FM performed the viral quantitation assays. All authors discussed the results and commented on the manuscript.

## Additional Information

**How to cite this article**: Ahmad, M.A., Mustafa, F., Ali, L.M., Karakkat, J.V. & Rizvi, T.A. Label-Free Capacitance-Based Identification of Viruses. *Sci. Rep.* **5**, 9809; DOI:10.1038/srep09809 (2015).

## Figures and Tables

**Figure 1:**
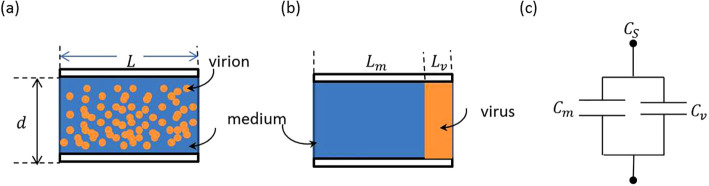
Electrical modeling of a virus suspension: (a) virion distribution in medium (b) two-zone model representation, and (c) the corresponding electrical equivalent capacitance model. *L* is length of the total capacitor; *d* is diameter of the capacitor; *L_m_* and *L_v_* are lengths of the medium and the viral zones, respectively; *C_s_, C_m_,* and *C_v_* are capacitance of the solution, medium, and virus, respectively.
